# Persistent *Chlamydia Pneumoniae *serology is related to decline in lung function in women but not in men. Effect of persistent Chlamydia pneumoniae infection on lung function

**DOI:** 10.1186/1471-2466-10-44

**Published:** 2010-08-25

**Authors:** Thorarinn Gislason, Vilmundur Guðnason, Bryndis Benediktsdottir, Isleifur Olafsson, Thor Aspelund, Bjarni Thjodleifsson, Christer Janson

**Affiliations:** 1 University of Iceland, Faculty of Medicine, Reykjavik, Iceland; 2 Department of Medicine Landspitali University Hospital, Reykjavik, Iceland; 3 Icelandic Heart Association, Kopavogur, Iceland; 4 Department of Clinical Biochemistry, Landspitali University Hospital, Reykjavik, Iceland; 5 Respiratory Medicine and Allergology, Uppsala University, Uppsala, Sweden

## Abstract

**Background:**

*Chlamydia pneumoniae *(C pn) infection causes an acute inflammation in the respiratory system that may become persistent, but little is known about the long-term respiratory effects of C pn infections. Aim: To estimate the long term respiratory effects of C pn with change in forced expiratory volume in one second (FEV_1_) and forced vital capacity (FVC) as a main outcome variable.

**Methods:**

The study comprised of 1109 subjects (500 men and 609 women, mean age 28 ± 6 years) that participated in the Reykjavik Heart Study of the Young. Spirometry and blood samples for measurements of IgG antibodies for C pn were done at inclusion and at the end of the follow-up period (mean follow-up time 27 ± 4 years).

**Results:**

Having IgG against C pn at both examinations was significantly associated to a larger decrease in FEV_1 _(6 mL/year) and FVC (7 mL/year) in women but not in men. In women the association between C pn and larger FEV_1 _decline was only found in women that smoked at baseline where having C pn IgG was associated with 10 mL/year decline compared to smokers without C pn IgG. These results were still significant after adjustment for age, smoking and change in body weight.

**Conclusion:**

Our results indicate that persistent C pn serology is related to increased decline in lung function in women but not in men. This effect was, however, primarily found in smoking women. This study is a further indication that the pathophysiological process leading to lung impairment may differ between men and women.

## Background

*Chlamydia pneumoniae *(C pn) is an intracellular gram-negative pathogen that is detected in 5 to 10% of community-acquired pneumonia and other lower respiratory tract infections [1] Most adults are infected at least once during their lifetime, as indicated by seroprevalence of 70 to 80% [2]. C pn respiratory diseases may manifest as an acute disease or persistent and recurring infection that causes intense chronic inflammation. Growing evidence indicates that inflammation results from cellular responses by non immune cells, including mucosal epithelial and vascular endothelial cells [3]. Studies have suggested that C pn may be related to the pathogenesis of wheeze in children [4], asthma in adults [5] and to chronic obstructive pulmonary disease (COPD) [6]. Systemic aspects of COPD include oxidative stress and altered circulating levels of inflammatory mediators and acute-phase proteins. C-reactive protein (CRP) reflects the total systemic burden of inflammation in several disorders and has been shown to up regulate the production of proinflammatory cytokines [7]. Systemic inflammation is increasingly being recognised as a risk factor for a number of different complications including atherosclerosis, cachexia, anorexia, and osteoporosis, but all of these complications are commonly observed in patients with COPD [8]. Associations between C pn serology and atherosclerosis [9] and ischemic heart diseases have been reported, as well as an additive or synergistic effect of other persistent infections on atherosclerosis [10]. These cardiovascular associations with persistent infections may be highly relevant in COPD since ischemic heart disease and stroke are the leading causes of mortality among patients with COPD [8].

Several studies have reported gender differences in the association between risk factors and pulmonary diseases. In one study of young children a positive serology for C pn was related to wheeze in girls but not in boys [4]. Other studies have shown gender differences in the association between various other risk factors and changes in lung function such as smoking [11] and CRP [12]. Most previous studies on the association of C pn and respiratory diseases have been performed on patient samples from different clinical settings, but large population-based longitudinal studies are lacking. No studies are available on the association between C pn serology and long term changes in lung function.

The primary aim of the present research was to study the association between C pn serology and changes in lung function in a longitudinal population study with particular focus on gender differences.

## Methods

### The Reykjavik Study of the Young

The Reykjavík Study of the Young was conducted in 3 stages between 1973 and 2003 and recruited 2147 participants aged 25-62 years. Stages 1,2 and 3 took part in the years: 1 (1973-1974); 2 (1983-1985); 3 (2001-2003). The present research is based on a subset of the Reykjavik Study of the Young which consisted of a random sample of individuals born in 1940 through 1954 who were living in the greater Reykjavik in 1973. The aim of the study was to compare younger generations to the older ones who were being investigated in the Reykjavik Study [12,13]

The present study focuses on the pulmonary part of the database, the methodology of which has been described separately [13]. Only subjects who had participated both at baseline (either stage 1 or 2) and follow-up (stage 3) were included in the present analysis. In addition two serum samples and acceptable pulmonary testing were needed. Altogether 1109 came for the follow-up study in 2001-2003, of whom 962 had come for the first stage in 1973-74 and 147 in 1983-85. The lung function tests included measurements of forced expiratory volume in one second (FEV_1_) and forced vital capacity (FVC).

Smoking status was recorded at each survey. Participants were divided into never smokers at baseline, current or ex-smokers, and the number of pack years was calculated. Body mass index (BMI) was calculated as weight in kilograms divided by the square of height in meters.

### Serologic methods

IgG antibodies against *Chlamydia pneumoniae *(C pn) were measured using reagents from the Immuno Biological Laboratories, Hamburg, Germany. Serum samples that gave indefinite results in antibody measurements were classified as seronegative.

### Statistics

Statistical analyses were performed using STATA 9 software (Stata Corp., Texas, USA). The Chi ^2 ^test and unpaired t test were used to compare characteristics between men and women. Multiple linear regression was used to analyse the association between lung function and C pn IgG status. A p-value < 0.05 was considered as statistically significant. In these regression analyses change in FEV_1 _and FVC from baseline to follow up (mL/year) was used as the dependent variable. The analyses were stratified by sex and the following independent variables were included: age (mean age between the surveys), age^2^, height, BMI, change in BMI and pack years between the surveys.

### Ethics

Approval was obtained from The National Bioethics Committee and The Data Protection Authority in Iceland

## Results

**Figure 1:**
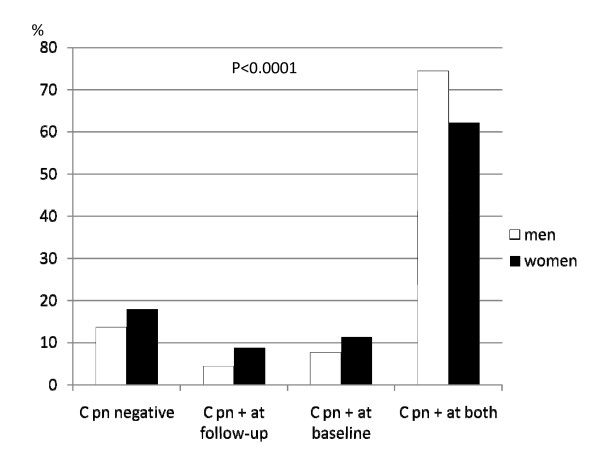
**Proportion men and women divided after Chlamydia pneumoniae (C pn) serology at baseline and follow-up**.

The characteristics of the study population are presented in Table 1. Women were more likely to be smokers at baseline and had a higher smoking exposure during the follow-up. Women were leaner at baseline but had a larger increase in BMI during the follow-up period. Men were more likely to have IgG antibodies against C pn at both surveys (Figure 1).

**Table 1:** Characteristics of the participants (% and mean ± SD)

	Men (n = 500)	Women (n = 609)	p-value	All (n = 1109)
Age at baseline	27.9 ± 5.7	27.4 ± 5.4	0.15	27.6 ± 5.6

Smoking history (%)			< 0.0001	

never	41.8	41.5		41.7

ex	23.6	14.1		18.4

current	34.6	44.3		40.0

Pack years between surveys in current smokers	14.7 ± 13.1	17.0 ± 12.5	0.04	15.8 ± 12.9

BMI kg/m2	23.2 ± 2.8	22.0 ± 3.1	< 0.0001	22.5 ± 3.0

Change in BMI over 10 yrs.	1.5 ± 1.2	1.9 ± 1.3	< 0.0001	1.7 ± 1.3

### C pn serology and change in lung function

Women with C positive C pn serology at both examinations had a larger decline in FEV_1 _and FVC than women with negative C pn serology, while no corresponding association was found in men. Women that developed positive C pn serology during the follow-up had a significantly larger decline in FVC, whereas the corresponding association to FEV_1 _was of border-line significance (p = 0.06) (Table 2). Decline in lung function was associated with an increase in BMI and smoking in both men and women, while a higher baseline BMI was associated with a greater decline in FVC but not in FEV_1._

**Table 2:** Association between C pn serology and change in FEV1 and FVC (mL/year) in men and women.

	Men		Women	
	**FEV1**	**FVC**	**FEV1**	**FVC**

C pn only at follow up	-1.8 (-11, 7.5)	-7.0 (-16, 2.5)	-5.6 (-11, 0.3)	-7.4 (-12, -2.6)

C pn only at baseline	2.6 (-5.0, 10)	-1.4 (-9.1, 6.3)	-0.5 (-5.9, 5.0)	-1.6 (-6.0, 2.8)

C pn at both examinations	2.1 (-2.9, 7.0)	-0.3 (-5.3, 4.8)	-4.5 (-8.2, -0.7)	-4.4 (-7.5, -1.3)

BMI at baseline (per 5 units)	0.6 (-2.6, 3.9)	-5.1 (-8.5,-1.8)	-1.1 (-3.5, 1.3)	-4.3 (-6.2, -2.3)

ΔBMI (per 10 years)	-2.1 (-3.5, -0.4)	-5.3 (-6.9, -3.7)	-2.2 (-3.2, -1.1)	-2.8 (-3.6, -1.9)

Pack years between surveys (per 10 units)	-2.1 (-3.5, -0.7)	-3.0 (-4.4, -1.5)	-1.6 (-2.7, -0.5)	-2.0 (-2.9, -1.0)

The estimates are adjusted for the variables in the table and age, height and BMI.

### Interactions

**Figure 2:**
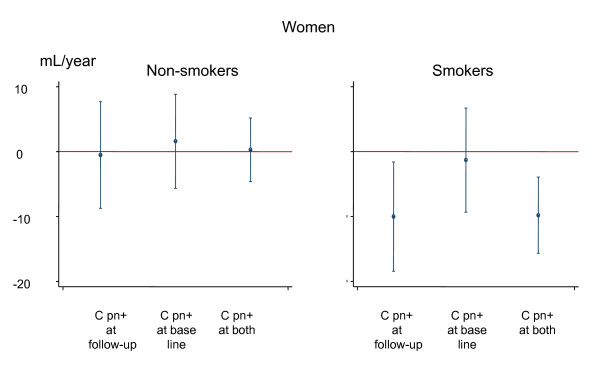
**Association between Chlamydia pneumoniae (C pn) serology and change in FEV_1 _(mL/year) in women that are non-smokers or current smokers**. The estimates are adjusted for age, height, smoking (pack years), BMI and change in BMI. Participants that were C pn negative at both surveys are the reference group in each smoking status group.

**Figure 3:**
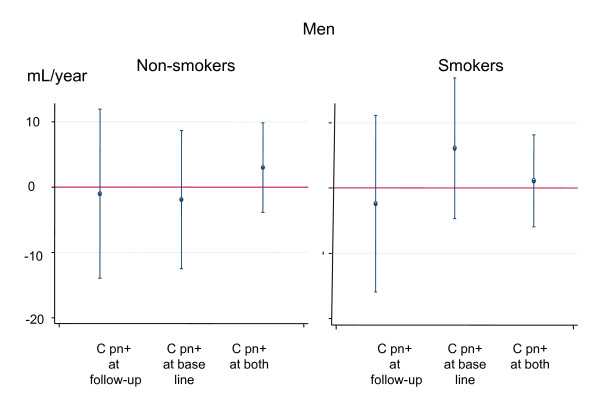
**Association between Chlamydia pneumoniae (C pn) serology and change in FEV_1 _(mL/year) in men that are non-smokers or current smokers**. The estimates are adjusted for age, height, smoking (pack years), BMI and change in BMI. Participants that were C pn negative at both surveys are the reference group in each smoking status group.

The sex difference in association between change in lung function and C pn serology was statistically significant for FEV1 (p_interaction _= 0.04) and almost significant for FVC (p_interaction _= 0.08). A significant interaction was found between women that were non-smokers and smokers at baseline (p_interaction _= 0.02). Women that were smokers had a significantly larger decline in lung function if they had a positive C pn serology at the second or at both examinations (Figure 2). No such corresponding interaction was found for men (Figure 3). No interactions were found concerning the association between C pn and FVC. No interactions were found for BMI or birth cohort concerning the association between C pn and change in lung function.

## Discussion

The main result of the study was that C pn infections were associated with increased lung function decline. This finding was, however, only seen in women. This study thereby provides further evidence of sex differences in the mechanisms related to decline in lung function. Our study also indicates that C pn may enhance the lung damaging effect of smoking in women.

The main strength of our study is that it is a population-based longitudinal study with a follow-up of 27 years. A potential weakness is that IgG antibodies measured by ELISA were used as the only indicator of persistent infection with C pn. Previous studies have shown that high levels of IgG antibodies to C pn measured by ELISA do not persist for half a decade after seroconversion without reinfection or reactivation [14]. In young military recruits IgG antibody levels measured by microimmunofluorescence test decrease rapidly after the infection [15]. The microimmunofluorescence test is however not suitable for seroepidemiological studies. Two studies have demonstrated that the ELISA test has comparable sensitivity and specificity to the microimmunofluorescence technique [14,16]. Another possible weakness is that our study population was not assessed by post-bronchodilator lung function testing and we can not know how much of the decline in FEV1 was due to reversible (asthma) or irreversible airflow obstruction (COPD). The widespread use of postbronchodilator spirometry is mostly confined to the twenty-first century. The long follow-up time of our study population is, however, unique.

The finding that C pn infections are more strongly related to lung impairment in women than in men fit surprisingly well with data from children where an association between wheeze and C pn IgG was stronger in girls than in boys [4]. There are several reports on gender differences in the association between wheezing and asthma irrespective of C pn or other infections. The predominant trend reported is a greater incidence of wheezing and asthma in boys with a reversal between ages 10-20 when the incidence becomes greater in females [17-22]. Girls are reported to be more vulnerable than boys to the impact of smoking and overweight on respiratory symptoms and lung function [23]. In contrast to the findings in children, Chinn et al found that weight gain had a larger effect in men than in women [11]. A stronger association between systemic inflammation and lung function decline in men than in women has also been reported in several studies [12,24,25]. Several mechanisms have been suggested to explain these gender differences. The sexes may develop their airway disease based on different sex related genetics[26] or on different immunological time scales [27]. Female sex hormones may also be part of the explanation for the gender difference as there is increasing evidence that sex hormones play a role in lung function development and decline. For an instance, it has been recently bee reported that girls with an early menarche have lower lung function as adults [28].

There are reports on a positive association of C pn and IgA and IgG serology and COPD [29,30] but negative associations have also been reported [31]. A causal association between C pn and COPD has, therefore, not been proven and possible mechanisms are not clear[32]. In a recent experimental study an intranasal inoculation with *C. pneumoniae *on day 0 was from day 7 associated with both sustained bronchial hyper-responsiveness and airway inflammation in mice [33]. An association between bronchial hyperresponsiveness and IgA antibodies against C pn has also been found in Swedish population study [34]. Acute *in vitro *experiments of human lung tissue with C pn suggest that C pn plays different roles during acute and chronic stages of pulmonary infection [35].

If persistent C pn infections play a significant role in the pathogenesis of asthma, COPD and lung function decline, this opens the therapeutic possibility of useful antibiotic treatment The data supporting antibiotic therapy are limited, however, as shown in a Cochrane review of macrolide usage in treatment of chronic asthma [36] and further long term studies are needed to confirm the possible role of persistent infections in the decline in lung function.

## Conclusion

In conclusion, our results indicate that persistent C pn serology is related to increased decline in lung function in women but not in men. This effect was, however, primarily found in smoking women. This study is a further indication that the pathophysiological process leading to lung impairment may differ between men and women.

## Abbreviations

BMI: body mass index; COPD: *chronic obstructive pulmonary disease*; C pn: *Chlamydia pneumoniae *; FEV1: forced expiratory volume in one second; FVC: forced vital capacity (FVC); IgA: immunoglobulin A; IgG: immunoglobulin G

## Competing interests

The authors declare that they have no competing interests.

## Authors' contributions

TG, BT and BB drafted the manuscript. VG was responsible for the clinical investigation, and data collection. IO carried out the serological measurements. TA was responsible for data management. CJ drafted the manuscript and carried out the statistical analyses. All authors read and approved the final manuscript.

## Pre-publication history

The pre-publication history for this paper can be accessed here:

http://www.biomedcentral.com/1471-2466/10/44/prepub
